# The complete chloroplast genome of *Cerasus dictyoneura*, an ornamental plant of China

**DOI:** 10.1080/23802359.2019.1673236

**Published:** 2019-10-07

**Authors:** Jing Liu, Rong Yin, Zhe Xu, Ying Tian

**Affiliations:** a State Key Laboratory of Seeding Bioengineering, Ningxia Forestry Institute, Yinchuan, Ningxia, China;; b School of Agriculture, Ningxia University, Yinchuan, Ningxia, China

**Keywords:** *Cerasus dictyoneura*, chloroplast genome, Illumina sequencing, phylogenetic analysis

## Abstract

The complete cp genome of *Cerasus dictyoneura* was 158,084 bp in length, consisting of a LSC of 86,275 bp and a SSC of 19,039 bp, which were separated by a pair of 26,385 bp IRs. The genome contained 131 genes, including 86 proteincoding genes, 37 tRNA genes, and 8 rRNA genes. The overall GC content is 36.7%, while the corresponding values of the LSC, SSC, and IR regions are 34.6, 29.5, and 42.6%, respectively. Furthermore, phylogenetic analysis based on 16 complete cp genome sequences indicated that *C. dictyoneura* is within Eurosids I.

*Cerasus dictyoneura* (Diels) Yu et L., in the family of Rosaceae, is a bush fruit tree that is endemic to China. Its fruit was known as ‘Calcium fruit’ (Kuang and Lu 1978). It is mainly distributed in the Shanxi, Shaanxi, Gansu, Ningxia and Henan province of China. For a long time, many plant resources of *Cerasus* were not highly valued in China. In recent years, with the development of fruit and flower tourism industry, a large number of cherry fruit and horticultural varieties were drawn from Japan, Europe and other places. The cognition, development and protection of native *Cerasus* resources are seriously lagged behind, and even mistaken for *Cerasus serrulata* originated in Japan, and *Cerasus pseudocerasus* originated in Europe (Yan et al. 2017). Therefore, exploring the classification of native *Cerasus* plant resources is beneficial to its effective cognition, development, and protection. *Cerasus dictyoneura* is an important *Cerasus* resource in China. But genome information of *C. dictyoneura* has been poorly studied.

Leaf samples of *C. dictyoneura* were collected from Yinchuan Botanical Garden (38ere collected E; Ningxia, NW China), and the specimens (CD3325) were deposited in the herbarium of state key laboratory of seeding bioengineering, Ningxia Forestry Institute, number is 2008PC0823.Genomic DNAs were extracted using a modified CTAB method (Doyle and Doyle 1987), quantified and further sequenced on the Illumina Hiseq Xten Platform (Illumina, San Diego, CA). The filtered reads were assembled using the program NOVOPlasty (Dierckxsens et al. 2017). The assembled chloroplast (cp) genome was annotated using Plann (Huang and Cronk 2015), and the annotation was corrected using Geneious R8.0.2 (Biomatters Ltd., Auckland, New Zealand). The map of the complete cp genome was generated using the web-based tool OGDRaw v1.2 (http://ogdraw.mpimp-golm.mpg.de/) (Lohse et al. 2013). The complete cp genome sequence has been submitted to GenBank under the accession number *MN259191.* The complete cp genome of *C. dictyoneura* is 158,084 bp long, and consists of two inverted repeat (IR) regions of 26,385 bp each, a large single-copy (LSC) region of 86,275 bp, and a small single-copy (SSC) region of 19,039 bp. The new sequence consists of 131 genes, including 86 protein-coding genes, 37 tRNA genes, and 8 rRNA genes. Intron–exon structure analysis indicated the majority (113 genes) are genes with no introns, whereas 16 genes contain a single intron and 2 protein-coding genes harbour two introns. The overall GC-content of the whole plastome is 36.7%, while the corresponding values of the LSC, SSC, and IR regions are 34.6, 29.5, and 42.6%, respectively.

To investigate the phylogenetic position of *C. dictyoneura*, a neighbour-joining (NJ) phylogenetic tree (Figure 1) was made based on the concatenated coding sequences of cp PCGs for 57 plastid genomes from published species of Rosaceae using MEGA7 with 1000 bootstrap replicates (Kumar et al. 2016) (http://www.megasoftware.net/). The result of the phylogenetic analysis shows that *C. dictyoneura*, is closely related to the species of *Cerasus humilis*. The complete cp genome sequence adds valuable information for the study of the genetic diversity of *C. dictyoneura*, and Rosaceae.

**Figure 1.**
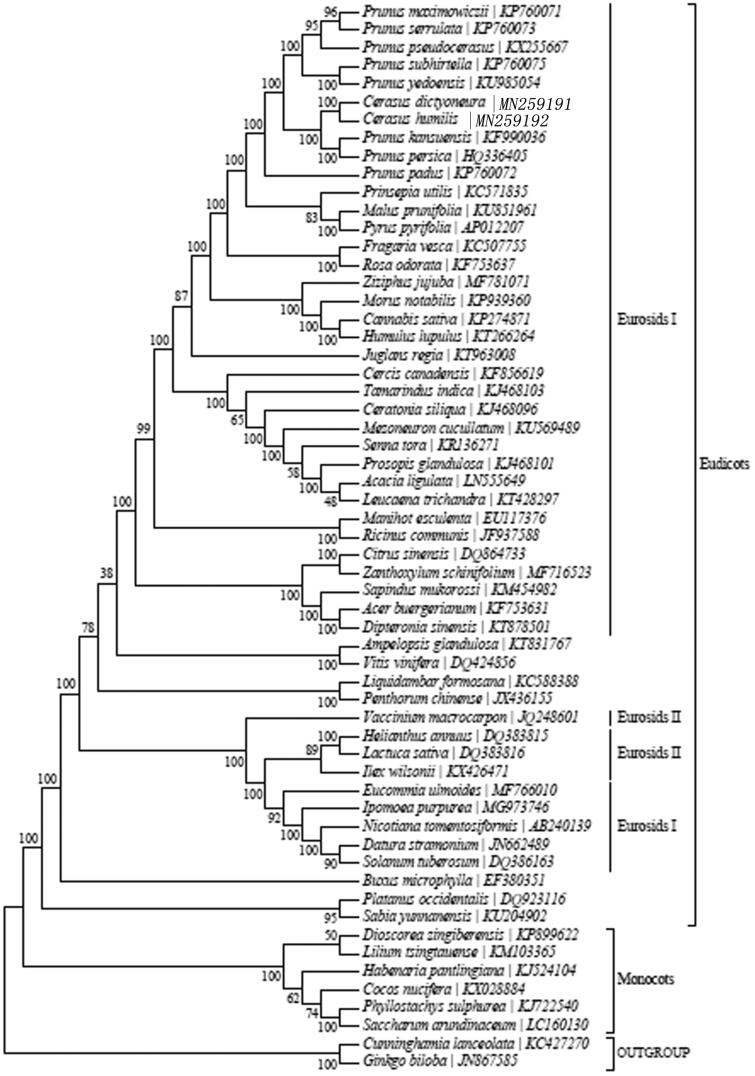
Maximum-likelihood (ML) tree of *C. dictyoneura* and its related relatives based on the complete chloroplast (cp) genome sequences.
